# Ischemia-Reperfusion Injury and Anesthesia

**DOI:** 10.1155/2014/980318

**Published:** 2014-06-19

**Authors:** Alexander Zarbock, Ahmet Eroglu, Engin Erturk, Can Ince, Martin Westphal

**Affiliations:** ^1^Department of Anesthesiology, Intensive Care and Pain Medicine, Münster University Hospital, Albert-Schweitzer-Campus 1, Building A1, 48149 Münster, Germany; ^2^Department of Anesthesiology and Intensive Care Medicine, Faculty of Medicine, Karadeniz Technical University, 61000 Trabzon, Turkey; ^3^Department of Intensive Care Adults, Erasmus University Medical Center, 3000 CA Rotterdam, The Netherlands; ^4^Fresenius Kabi Aktiengesellschaft, Bad Homburg vor der Höhe, Germany

Ischemia-reperfusion injury represents a pathological condition characterized by an initial undersupply of blood to an area or organ followed by a restoration of perfusion and concomitant reoxygenation (= reperfusion). Ischemia typically occurs in the presence of embolism or thrombosis but can also be triggered by surgery and transplantation. Anyway, the disturbance in perfusion results in a severe imbalance between metabolic supply and demand, subsequently causing tissue hypoxia [1]. Notably, these initial changes cause time-dependent molecular and structural alterations. In this context, it is also important to consider that all tissues and organs are susceptible to ischemia, but susceptibility to an ischemic insult differs between organ systems. Whereas the brain can endure ischemia only a few minutes, other tissues (e.g., muscle) are able to withstand ischemia for a long time without signs of irreversible damage.

Interestingly, restoration of blood flow and reoxygenation is commonly associated with an exacerbation of tissue injury and a profound inflammatory response (“reperfusion injury”) [1, 2]. Ischemia-reperfusion injury contributes to pathology in a wide range of conditions.

For example, myocardial ischemia followed by reperfusion typically manifests in microvascular dysfunction, death of myocytes, and myocardial stunning or dysfunction.

Ischemia-reperfusion injury (IRI) of the lung, for example, following transplantation, is characterized by nonspecific alveolar damage, edema formation, and hypoxemia. The clinical spectrum of pulmonary IRI may range from mild hypoxemia to acute respiratory distress syndrome.

In contrast to other organs, the brain is particularly susceptible to ischemia and irreversible neuronal damage already occurs after only 5 minutes of complete ischemia [3]. For brain ischemia, as occurring in the setting of stroke, reestablishing reperfusion seems to be only beneficial, if carried out within a short time period after the onset of ischemia. Reperfusion of ischemic stroke seems to be very critical, as patients may suffer from cerebral reperfusion injury manifesting in fatal cerebral edema formation and intracranial hemorrhage.

IRI of the kidney may occur in the setting of transplantation and cardiac arrest and during cardiac surgery. Here it is important to note that renal injury is usually associated with a high morbidity and mortality. The cortical-medullary region is the most susceptible region to tubular injury, inflammation, and vascular alterations.

Generally, IRI of a single organ causes the release of different proinflammatory mediators, which may subsequently induce inflammation in other organs, thereby potentially contributing to multiple organ dysfunction or even failure [4].

Different pathological processes contribute to tissue injury secondary to ischemia-reperfusion. During ischemia, limited oxygen availability leads to an impaired endothelial cell barrier function with a concomitant increase in vascular permeability and leakage due to decreases of intracellular cAMP levels caused by a reduced adenylate cyclase activity [1]. Furthermore, ischemia-reperfusion induces cell death due to apoptosis, necrosis, and autophagy [5]. During the ischemic period, alterations in the transcriptional control of gene expression likewise occur. Another mechanism implicated in the pathophysiology of injury during ischemia is the inhibition of oxygen-sensing prolyl hydroxylase (PHD) enzymes, because they require oxygen as a cofactor. Hypoxia-triggered inhibition of PHD enzymes induces the posttranslational activation of hypoxia and inflammatory signaling cascades, which in turn regulate the stability of the transcription factors, hypoxia-inducible factor (HIF) and nuclear factor-*κ*B (NF-*κ*B) [2].

Reperfusion of ischemic tissue activates a complex inflammatory response without the involvement of pathogenic triggers, a phenomenon also referred to as sterile inflammation. During the initiation of this inflammatory response, endogenous molecules act as alarmins or danger-associated molecular patterns (DAMPs) [6]. The inflammatory process is stimulated through self-antigens, which are functional components of intact cells but become stimulators of innate immunity when released from injured or dying cells [6]. In 1996, Weiser et al. discovered and described a novel mechanism for reperfusion injury that involves antibody deposition and activation of the complement system leading to an acute inflammatory response [7]. One decade later, the concept of innate autoimmunity was introduced, which is based on the discovery that circulating natural antibodies recognize self-antigens and elicit an acute inflammatory response involving the complement system [8]. Although ischemia-reperfusion is typically established in a sterile environment, activation of innate and adaptive immune responses occurs and contributes to injury, including activation of pattern-recognition receptors such as TLRs and inflammatory cell trafficking into the injured organ [9]. During this inflammatory process, the coagulation system is also activated, because the innate immune system and coagulation system are highly interconnected [10]. As ischemia-reperfusion injury is a common clinical problem and is associated with relevant complications, it is important to identify therapeutic approaches which prevent or at least mitigate ischemia-reperfusion-induced organ injury and organ dysfunction.

This special issue is devoted to the modulation of ischemia-reperfusion injury by different measures and contains eight original papers addressing this clinically relevant topic. These papers are accompanied by two review articles dealing with the effects of anesthetics on ischemia-reperfusion injury. Papers from B. U. Togrul et al., D. Dohman et al., and Y. Demirci et al. are focusing on ischemia-reperfusion injury of the liver. In two of these three papers, different therapeutic interventions on hepatic ischemia-reperfusion injury are evaluated, whereas the third paper is a retrospective study in which the authors investigated the efficacy and safety of intermittent portal triad clamping with low central venous pressure during liver resection. In this context, it has been reported that remote ischemic preconditioning and therapeutic interventions can reduce liver damage after inducing ischemia-reperfusion injury. The studies by S. C. Karahan et al., B. Michèle et al., S. C. Karahan et al., D. Dohman et al., and G. Altun et al. elucidate the effects of different anesthetic techniques and drugs on ischemia-reperfusion injury. These eight papers are entitled as follows:* “The effects of remote ischemic preconditioning and N-acetylcysteine with remote ischemic preconditioning in rat hepatic ischemia-reperfusion injury model”* by B. U. Togrul et al.,* “The effects of spinal, inhalation, and total intravenous anesthetic techniques on ischemia-reperfusion injury in arthroscopic knee surgery”* by S. C. Karahan et al.,* “Efficacy and safety of hepatectomy performed with intermittent portal triad clamping with low central venous pressure"* by D. Dohman et al.,* “Adalimumab ameliorates abdominal aorta cross clamping induced liver injury in rats”* by Y. Demirci et al.,* “Evidence for the use of isoflurane as a replacement for chloral hydrate anesthesia in experimental stroke: an ethical issue”* by B. Michèle et al.,* “The effect of dexmedetomidine on oxidative stress during pneumoperitoneum”* by S. C. Karahan et al.,* “The comparison of the effects of sevoflurane inhalation anesthesia and intravenous propofol anesthesia on oxidative stress in one lung ventilation”* by D. Dohman et al., and* “Role of ethyl pyruvate on systemic inflammatory response and lung injury in an experimental model of ruptured abdominal aortic aneurysm”* by G. Altun et al.

*Alexander Zarbock* *Ahmet Eroglu* *Engin Erturk* *Can Ince* *Martin Westphal*
